# Cognitive decline in acoustic neuroma patients: An investigation based on resting-state functional magnetic resonance imaging and voxel-based morphometry

**DOI:** 10.3389/fpsyt.2022.968859

**Published:** 2022-08-01

**Authors:** Xueyun Deng, Lizhen Liu, Zhiming Zhen, Quan Chen, Lihua Liu, Xuhui Hui

**Affiliations:** ^1^Department of Neurosurgery, The Affiliated Nanchong Central Hospital of North Sichuan Medical College, Nanchong, China; ^2^Department of Neurosurgery, West China Hospital of Sichuan University, Chengdu, China; ^3^Department of Radiology, Southwest Hospital, Third Military Medical University (Army Medical University), Chongqing, China; ^4^Department of Neurology, Chenjiaqiao Hospital, Chongqing, China; ^5^Department of Geriatrics, The Affiliated Nanchong Central Hospital of North Sichuan Medical College, Nanchong, China

**Keywords:** cognition, acoustic neuroma, vestibular schwannoma, rs-fMRI, VBM

## Abstract

**Objective:**

Acoustic neuroma (AN) is a clinically common benign tumor. There are few neuropsychological investigations for AN, especially cognitive neuropsychology. Herein, the study probed into cognitive function changes in AN patients and expounded possible mechanisms through structural and functional magnetic resonance imaging (fMRI).

**Materials and methods:**

Neuropsychological tests were performed between 64 patients with AN and 67 healthy controls. Then, using resting-state fMRI, the possible mechanisms of cognitive decline in AN patients were further explored by calculating the amplitude of low-frequency fluctuations (ALFF) and regional homogeneity (ReHo). Furthermore, using high-resolution T1-weighted images, voxel-based morphometry (VBM) was adopted to investigate the changes in gray matter volume (GMV) and white matter volume (WMV) in AN patients.

**Results:**

AN patients had worse cognitive performance than those in the healthy controls. Relative to the healthy individuals, the mALFF value was increased in the right caudate nucleus of the patients with left-sided AN (LAN) and the right rectus region of the patients with right-sided AN (RAN). The mReHo values of the bilateral superior frontal gyrus and middle frontal gyrus were decreased in LAN patients. Compared with healthy subjects, the GMV values were elevated in the left fusiform gyrus, parahippocampal gyrus, calcarine gyrus, and cuneus in LAN patients as well as in the right fusiform gyrus and parahippocampal gyrus in RAN patients. Meanwhile, the WMV values showed elevations in the bilateral putamen, left rectal gyrus, and thalamus in LAN patients.

**Conclusion:**

Cognitive dysfunction occurs in AN patients. Cognitive decline in AN patients activates functional activity in some brain regions, thereby compensating for cognition decline. Additionally, the ReHo values were reduced in the frontal lobe in LAN patients, and the connectivity was decreased, affecting the functional differentiation and integration of the brain, which may be associated with the decline in cognitive function. Lateralized brain reorganization induced by unilateral hearing loss was presented in AN patients. LAN caused a more significant interference effect on the brain while RAN patients showed more stable cerebral cortices. Altogether, responding to cognition decline in AN patients, structural reorganization occurs, and compensative increases in cognitive-related brain regions, which compensates for cognitive impairment.

## Introduction

The functions of the brain are constantly changed and remodeled to functionally adapt to changing sensory signals (1, 2). Prior studies have demonstrated that the auditory center of deaf patients can be activated by non-auditory stimuli (3), which indicates cross-modal sensory plasticity in deaf patients (4). Plasticity can be also presented in the auditory cortex, with structural changes outside the auditory area (5–7). Hearing loss can affect different aspects of cognitive function in pre- and post-lingually deaf populations, such as attention deficit (8, 9), impairment in short-term memory (10), and executive function (11–13). There are at least two forms of brain plasticity during hearing impairment: cross-modal plasticity suggests that the body can compensate for hearing loss by optimizing multiple sensory systems (4), while changes in cognitive function suggest that more cognitive resources must be utilized during auditory processing to compensate for hearing loss (10, 14).

Unlike bilateral deafness, patients with unilateral hearing loss (UHL) retain most of their ability to capture auditory signals but have more complicated auditory processing (15–18). UHL shows asymmetric auditory input, which not only affects overall auditory perception (19) but also affects the processing of higher-order auditory representations (20–22). Therefore, it is reasonable to propose that the connectivity of sensors with advanced control networks and the integration between these networks may be functionally reorganized in the UHL patients.

Studies have illustrated the plasticity of the central auditory pathway in UHL, but most of these studies are limited to the auditory cortex and auditory pathway (23, 24). Up to now, few studies have investigated the relationship between UHL and cognitive function, particularly the mechanisms of cognitive decline. Whether unilateral auditory deprivation affects the neural circuits of cognitive control networks other than the sensory cortex remains unclear. Zhang et al. (25) enrolled 11 patients with left sensorineural hearing loss and 10 patients with right sensorineural hearing loss and 11 healthy individuals, and found differences in the regional homogeneity (ReHo) values in the default mode network (DMN), which is considered to be related to cognitive impairment. However, no significant differences were detected in the neuropsychological test scores (MMSE, TMTA, TMTB, etc.) among the three groups, which may be related to the small sample size and low statistical power, and MMSE is less sensitive than MoCA. Wang et al. (26) investigated 21 patients with left and 21 patients with right-sided acoustic neuroma and 24 healthy subjects and analyzed the gray matter volume (GMV) with voxel-based morphometry (VBM). Their results uncovered volume decreases in key brain regions for cognitive processing such as bilateral anterior cingulate cortex, right superior frontal gyrus, and bilateral middle frontal gyrus. They speculated that long-term partial hearing impairment could change the auditory cortex and affect higher-level cognitive function; whereas, no difference was noted in the MMSE score of the enrolled patients, suggesting no difference concerning cognitive function among the three groups, so the changes in white matter volume were not assessed.

Resting-state functional magnetic resonance imaging (rs-fMRI) is a promising imaging technique for the non-invasive detection of whole-brain functional activity. Amplitude of low-frequency fluctuations (ALFF), which indicates the intensity of local brain activity, and ReHo, which represents the synchrony of the blood-oxygen level-dependent (BOLD) signal adjacent to voxels over time, are two robust indicators showing high test-retest reliability (27, 28). It is quite valuable for exploring the neural basis of individual differences in sensory deprivation.

We hypothesized that the cognitive decline caused by patients with acoustic neuroma could lead to changes in ALFF and ReHo in related brain regions, as well as in the cerebral cortex involved in cognitive function. Therefore, this experiment was designed to analyze and calculate relevant indicators to identify the brain regions presenting differences and analyze their correlations with cognitive function, so as to find changes in brain function and cortical structural reorganization after cognitive impairment in patients.

## Materials and methods

### Demographics, clinical data, and cognitive function of subjects

Sixty-four right-handed AN patients (21 males and 43 females, age range: 19–75 years) were recruited from the outpatient and ward of Neurosurgery of West China Hospital of Sichuan University, from October 2019 to July 2020. Sixty-seven right-handed hearing controls (21 males and 46 females, age range: 26–74 years) were also enrolled. The demographic information is shown in Supplementary Tables 1, 2. The experiment was approved by the Hospital Ethics Committee, and all participants signed the informed consent.

The average air conduction thresholds at four frequencies (0.5, 1, 2, and 4 kHz) were calculated as the pure tone average (PTA), representing the hearing levels of the subjects. According to the World Health Organization, hearing loss was classified as mild (PTA 26–40 dB HL), moderate (PTA 41–60 dB HL), severe (PTA 61–80 dB HL), and profound (PTA > 81 dB HL).

Patients with acoustic neuroma are often complicated with tinnitus symptoms. In this study, AN patients accompanying tinnitus were assessed using the tinnitus handicap inventory (THI) scale (29). Higher scores indicate greater severity and greater impact on daily life.

All subjects were performed MoCA, RAVLT immediate memory and delayed memory, Stroop color-word test A, B, and C (Stroop A, B, and C), symbol digit modalities test (SDMT), trail making test A and B (TMT A and B).

### MRI scanning parameters

A GE 750 W 3.0 T magnetic resonance apparatus was used for 3D T1 image acquisition from all enrolled patients and healthy controls, and data were collected using a 32-channel head coil. rs-fMRI scanning parameters were set as follows: repetition time (TR) = 2,000 ms, echo time (TE) = 30 ms, field of view (FOV) = 24.0 cm × 24.0 cm, acquisition matrix = 64 × 64, flip angle = 90°, slice thickness/slice spacing = 4.0 mm/0.0 mm, voxel size = 3.75 × 3.75 × 4.0 mm^3^. In total, 35-slice whole-brain images were scanned inter-slice at 240 time points, with a scanning time of about 8 min and 10 s. The T1 scan parameters were as follows: slice thickness = 1 mm, TR = 8.68 ms, TE = 3.20 ms, scan matrix = 512 × 512, voxel size = 0.5 × 0.5 × 1.0 mm^3^, and the scan time is about 4 min and 37 s. The head of the patient was appropriately fixed with soft silicone on both sides to reduce the possibility of head displacement.

### Image data processing process

The rs-fMRI data were preprocessed, and ALFF and ReHo values were calculated using the Restplus software package (RESTplus V1.2, http://restfmri.net/forum/RESTplusV1.2). The data preprocessing process is as follows: (1) removal of volumes at the first 10 time points; (2) followed by slice timing and realign. The translational head movement should not exceed 2 mm and increased to within 3 mm for very few subjects who are easy to agitate due to dementia, diseases, and other reasons; (3) normalization: one-step registration method; (4) detrend; (5) regression of covariates, such as white matter, cerebrospinal fluid, and head movement signals to reduce their influence on the experiment; (6) Filter; and (7) Smooth.

It should be noted that ALFF was calculated without filtering during the preprocessing process, and ReHo was not smoothed during the preprocessing but smoothed after it was calculated, to allow the data to be normalized, which would be conducive to statistical analysis and indicator standardization; the mean ALFF (mALFF) value was generated and mean ReHo (mReHo) value was obtained for later analysis.

#### VBM preprocessing process

Using MATLAB2013b (Math Works, Natick, MA, USA), the SPM8 software package (30) was run with the steps as follows: (1) Conversion of DICOM format images to 3D NIFTI format files; (2) origin-corrected anatomical image (3) Image tissue segmentation and spatial normalization. (4) Calculation of final volume after image segmentation. (5) Smoothing: within the range of the full width at half maximum (FWHM) of the Gaussian smoothing kernel of 6–10, 8 mm was selected in this study.

### Statistical analysis

Statistical analysis was performed using SPSS 23.0 (IBM). Continuous variables that met the normal distribution and homogeneity of variance were presented as the mean ± standard deviation, otherwise, the median (interquartile range) was used. Categorical variables were expressed as numerical values (percentages). Independent data between two groups were compared by the *t*-test if the data met the normal distribution and homogeneity of variance, otherwise, the non-parametric Mann-Whitney U test was used. The comparisons between the two acoustic neuroma subgroups and three groups (including healthy controls) were performed by one-way ANOVA if the data conformed to the normal distribution and homogeneity of variance, otherwise, by the Kruskal-Wallis H test. Qualitative data were compared using the χ^2^ test. Spearman correlation analysis was conducted to explore the correlations of clinical indicators with cognitive function, depression, and anxiety. A *p*-value < 0.05 was considered statistically significant.

The SPM12.0 software package was employed to perform an independent-sample *t*-test for the inter-group comparisons of left-sided and right-sided acoustic neuroma patients and healthy subjects. The analysis of covariance (ANCOVA) was adopted for intra-group comparisons of differences between the groups by limiting the scope of analysis, based on controlling age, gender, and years of education. The differential brain regions were made into a mask, followed by a *post-hoc* comparison within the range limited by this mask. An adjusted cluster-level test was conducted for statistical analysis of gray matter and white matter, and significance was defined as *p* < 0.001 at the voxel level, and family-wise error (FWE)-corrected *p*-value < 0.05 at the cluster level. The GMV and WMV values of differential brain regions were extracted and their correlations with cognitive function scale scores and clinical indicators were assessed by Spearman correlation analysis. *p* < 0.05 was considered to be a significant statistical difference.

## Results

### Results of demographics and clinical data comparisons among groups

The demographics and clinical characteristics of the subjects are presented in Table 1. In total, 131 subjects were enrolled in this study, including 64 patients with acoustic neuroma (left: right = 40: 24) and 67 healthy controls. We observed no significant difference in age, gender, and education level between the acoustic neuroma patients and healthy controls (*p* > 0.05). The results of the cognitive function scale and mental health scale of acoustic neuroma patients and healthy controls are outlined in Supplementary Tables 1, 2.

**Table 1:** Comparison of clinical data and cognitive function among LAN, RAN, and HC groups.

	**LAN (*n =* 40)**	**RAN (*n =* 24)**	**HC (*n =* 67)**	**F/H/T values**	***p*-value**	***Post-hoc***
Gender (male)	16 (40.0%)	5 (20.8%)	21 (31.3%)	2.562	0.278^a^	N/A
Age (yrs)	49.00 ± 14.32	50.50 ± 12.27	46.64 ± 10.06	1.105	0.334^b^	N/A
Years of education (yrs)	10.00 (7.80)	8.50 (8.30)	9.00 (7.00)	4.825	0.090^c^	N/A
Course of disease (yrs)	2.00 (5.23)	2.75 (5.25)	N/A	1.487	0.223^d^	N/A
THI	14.00 (6.00)	10.00 (6.00)	N/A	0.221	0.639^d^	N/A
Left PTA (dB HL)	55.73 ± 26.14	22.15 ± 10.67	N/A	6.304	<0.001^e^^*^	N/A
Right PTA (dB HL)	17.50 (10.00)	66.74 ± 34.06	N/A	−6.031	<0.001^d^^*^	N/A
MoCA scores	21.00 (6.00)	19.00 (9.00)	26.00 (5.00)	37.407	<0.001^c^^*^	RAN < HC LAN < HC
RAVLT immediate recall	34.00 (16.00)	31.00 (15.00)	47.00 (19.00)	31.927	<0.001^c^^*^	RAN < HC LAN < HC
RAVLT delay recall	6.28 ± 3.52	5.29 ± 2.97	9.06 ± 3.32	15.404	<0.001^b^^*^	RAN < HC LAN < HC
Stroop A (s)	32.50 (27.50)	36.00 (17.50)	27.00 (12.50)	10.053	0.007^c^^*^	RAN>HC
Stroop B (s)	48.50 (33.00)	54.00 (32.00)	37.00 (19.00)	16.996	<0.001^c^^*^	RAN>HC LAN>HC
Stroop C (s)	125.50 (85.00)	131.00 (64.00)	86.00 (52.00)	22.064	<0.001^c^^*^	RAN>HC LAN>HC
SDMT	35.36 ± 15.66	31.38 ± 17.06	45.16 ± 16.84	8.574	0.004^b^^*^	RAN < HC LAN < HC
TMT A (s)	52.00 (68.00)	73.50 (62.00)	38.00 (31.00)	19.890	<0.001^c^^*^	RAN>HC LAN>HC
TMT B (s)	180.50 (190.00)	231.00 (191.00)	104.00 (111.00)	16.473	<0.001^c^^*^	RAN>HC LAN>HC
HAMD	8.00 (8.00)	10.00 (5.00)	2.00 (3.00)	65.865	<0.001^c^^*^	RAN>HC LAN>HC
HAMA	6.00 (6.00)	7.00 (6.00)	2.00 (2.00)	61.010	<0.001^c^^*^	RAN>HC LAN>HC

a p and

b p-values were obtained by the R × C chi-square test and ANOVA test, respectively.

c p and

d p-values were obtained by Kruskal-Wallis and Mann-Whitney (non-parametric test), respectively.

e p-values obtained by t-test. F values, H values, and T values were obtained by ANOVA, Kruskal-Wallis, and t-test, respectively. All data were expressed as mean ± SD, median (interquartile range), or number (percentage). The significance level was set at p < 0.05.

* p < 0.05. LAN, left acoustic neuroma; RAN, right acoustic neuroma; HC, healthy controls; PTA, pure tone average; THI, tinnitus handicap inventory; N/A, not available.

The cognitive function and mental statuses were compared among patients with left-sided and right-sided acoustic neuroma and healthy controls. There were no noticeable differences concerning gender, age, and years of education among the three groups (*p* > 0.05). As depicted in Table 1, no significant difference was noted in the course of disease and THI score between the left-sided and right-sided acoustic neuroma patients (*p* > 0.05). As compared to healthy controls, patients with left-sided acoustic neuroma showed significantly poorer performance in MoCA score, RAVLT immediate memory and delayed memory, Stroop B, Stroop C, SDMT, TMT A, and TMT B (*p* < 0.05), while patients with right-sided acoustic neuroma displayed notably poorer performance in MoCA score, RAVLT immediate memory and delayed memory, Stroop A, Stroop B, Stroop C, SDMT, TMT A, and TMT B (*p* < 0.05). The results are summarized in Tables 1, 2.

**Table 2:** Comparison of MoCA among LAN, RAN, and HC groups.

	**LAN** **(*n =* 40)**	**RAN (*n =* 24)**	**HC (*n =* 67)**	**H values**	***p-*value**	***Post-hoc***
Visuospatial executive	3.00 (2.00)	2.00 (2.00)	4.00 (2.00)	44.844	<0.001^*^	RAN < HC LAN < HC
Naming	3.00 (1.00)	2.00 (2.00)	3.00 (1.00)	10.569	0.005^*^	RAN < HC RAN < LAN
Attention	5.00 (1.00)	5.00 (1.00)	6.00 (0.00)	27.776	<0.001^*^	RAN < HC LAN < HC
Language	1.50 (1.00)	1.00 (2.00)	2.00 (2.00)	18.815	<0.001^*^	RAN < HC LAN < HC
Language: sentence repetition	1.00 (1.00)	0.00 (1.00)	1.00 (1.00)	15.045	0.001^*^	RAN < HC LAN < HC
Language: fluency task	1.00 (0.00)	1.00 (1.00)	1.00 (0.00)	14.677	0.001^*^	RAN < HC LAN < HC
Abstract thinking	1.00 (2.00)	1.00 (2.00)	2.00 (1.00)	9.391	0.009^*^	RAN < HC
Delayed recall	2.00 (2.00)	2.00 (2.00)	3.00 (3.00)	13.884	0.001^*^	RAN < HC LAN < HC
Orientation	6.00 (1.00)	6.00 (1.00)	6.00 (0.00)	12.543	0.002^*^	RAN < HC LAN < HC
MoCA scores	21.00 (6.00)	19.00 (9.00)	26.00 (5.00)	37.407	<0.001^*^	RAN < HC LAN < HC

p- and H values were obtained by Kruskal-Wallis H (non-parametric test). Data were expressed as median (interquartile range).

* p < 0.05. LAN, left acoustic neuroma; RAN, right acoustic neuroma; HC, healthy controls; MoCA, Montreal cognitive assessment.

Patients with acoustic neuroma are often complicated with tinnitus symptoms. Correlation analysis showed that there was no significant correlation between THI and cognitive scale, or THI and anxiety, depression scale.

### Comparisons of ALFF and ReHo values of rs-fMRI among groups

**Figure 1:**
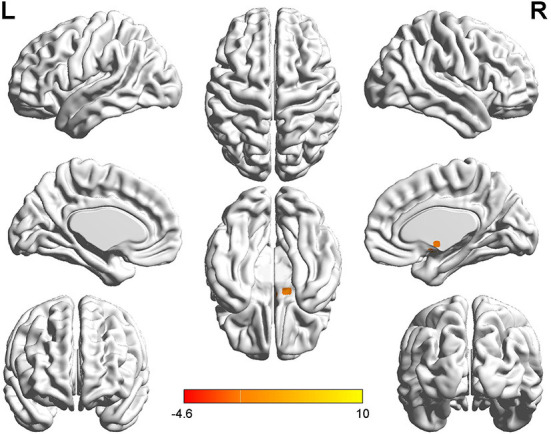
*T*-value map of differential results of mALFF between left auditory neuroma and healthy controls. Compared with healthy controls, the value of mALFF in the right caudate nucleus of patients with left acoustic neuroma was increased, and there was no significant decrease in the brain regions. The statistical threshold was voxel-wise *p* < 0.001 with cluster-wise FWE corrected *p* < 0.05 (122 voxels).

**Figure 2:**
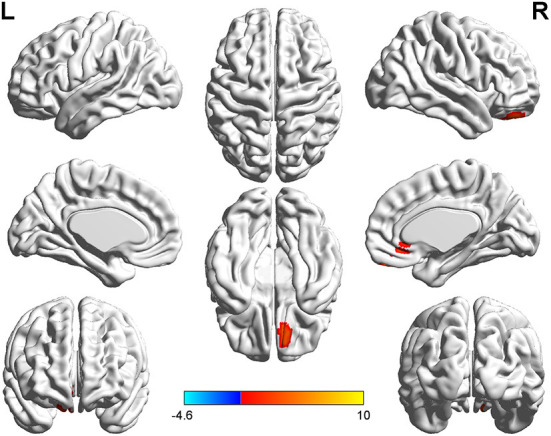
*T*-value map of differential results of mALFF between right auditory neuroma and healthy controls. Compared with healthy controls, the value of mALFF in the right rectus gyrus of patients with right acoustic neuroma was increased, and there was no significant decrease in the brain regions. The statistical threshold was voxel-wise *p* < 0.001 with cluster-wise FWE corrected *p* < 0.05 (122 voxels).

**Figure 3:**
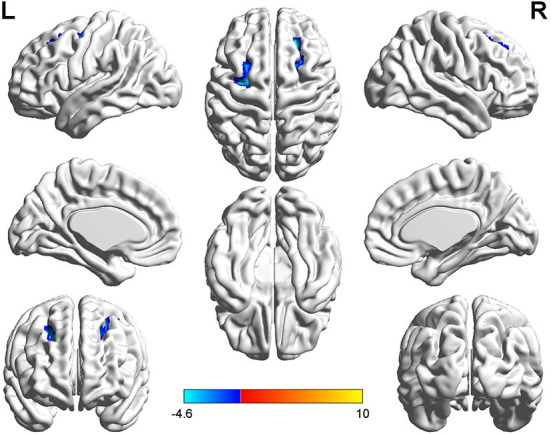
*T*-value map of differential results of mReHo between left auditory neuroma and healthy controls. Compared with healthy controls, the brain regions with decreased mReHo in the patients with left acoustic neuroma were: the bilateral superior and middle frontal gyrus, with no significant decrease in the brain regions. The statistical threshold was voxel-wise *p* < 0.001 with cluster-wise FWE corrected *p* < 0.05 (122 voxels).

Relative to healthy controls, the mALFF values in the right caudate nucleus and right thalamus were increased in patients with left-sided acoustic neuroma and the right rectal gyrus in patients with right-sided acoustic neuroma; reversely, the mReHo values in bilateral superior frontal gyrus and middle frontal gyrus were detected in patients with left-sided acoustic neuroma, while no obvious change was found in the patients with right-sided acoustic neuroma (Figures 1–3, Supplementary Figure 1).

### Comparisons of GMV and WMV values of VBM

**Figure 4:**
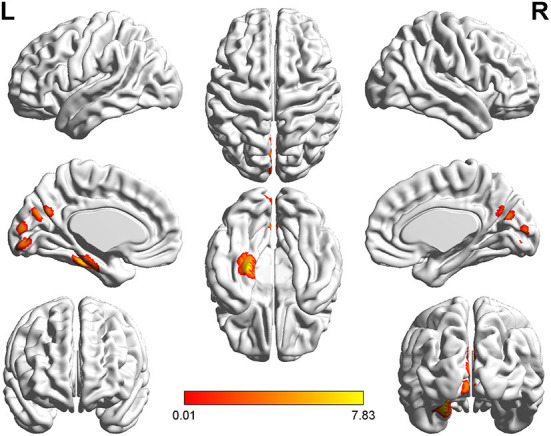
*T*-value map of increased gray matter volume in the patients with left acoustic neuroma compared with healthy controls. Compared with healthy controls, the brain regions with increased gray matter volume in the patients with left acoustic neuroma were: the left parahippocampal gyrus, the fusiform, the calcarine, and the cuneus. The statistical threshold was voxel-wise *p* < 0.001 with cluster-wise FWE corrected *p* < 0.05 (487 voxels).

**Figure 5:**
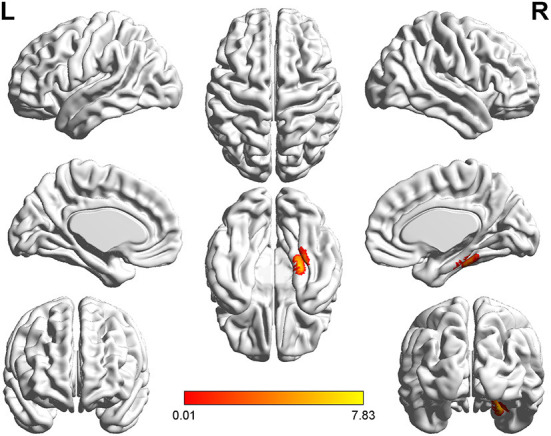
*T*-value map of increased gray matter volume in the patients with right acoustic neuroma compared with healthy controls. Compared with healthy controls, the brain regions with increased gray matter volume in the patients with right acoustic neuroma were: the right fusiform and parahippocampal gyrus. The statistical threshold was voxel-wise *p* < 0.001 with cluster-wise FWE corrected *p* < 0.05 (513 voxels).

**Figure 6:**
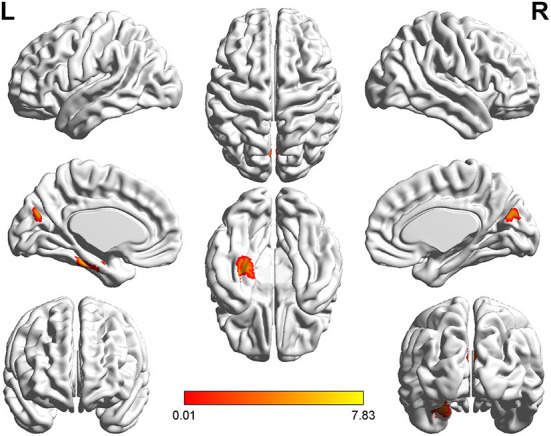
*T*-value map of increased gray matter volume in the patients with left acoustic neuroma compared with right acoustic neuroma. Compared with the patients with right acoustic neuroma, the brain regions with increased gray matter volume in the patients with left acoustic neuroma were: the left parahippocampal gyrus and fusiform. The statistical threshold was voxel-wise *p* < 0.001 with cluster-wise FWE corrected *p* < 0.05 (319 voxels).

**Figure 7:**
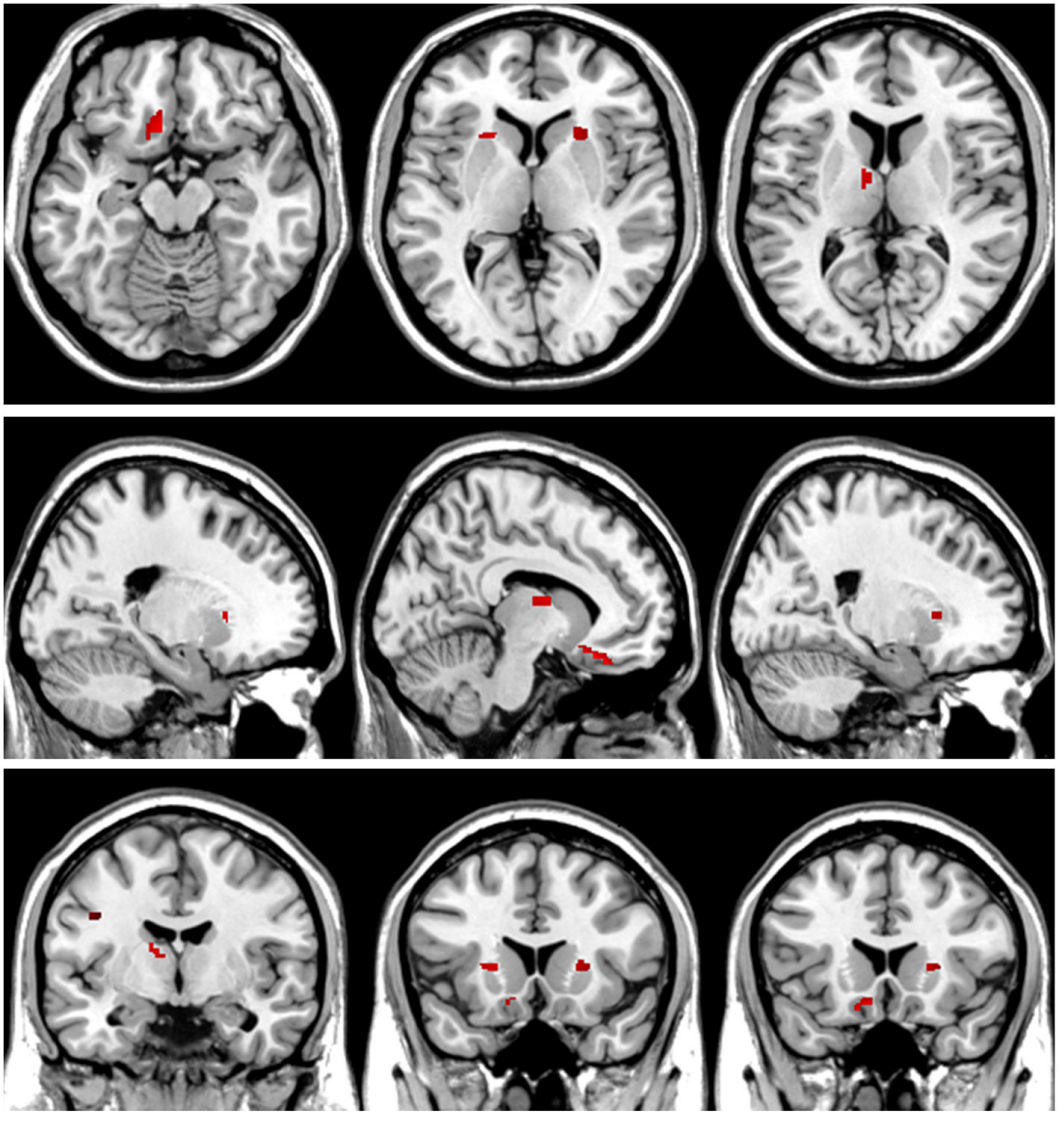
*T*-value map of increased white matter volume in the patients with left acoustic neuroma compared with healthy controls. Compared with the healthy controls, the brain regions with increased white matter volume in the patients with left acoustic neuroma were: the bilateral putamen, left rectus, and left thalamus. The statistical threshold was voxel-wise *p* < 0.001 with cluster-wise FWE corrected *p* < 0.05 (28 voxels).

The patients with left-sided acoustic neuroma showed increases in the GMV values of the left fusiform gyrus, parahippocampal gyrus, calcarine gyrus, and cuneus while the patients with right-sided acoustic neuroma exhibited elevations in the GMV values of the right fusiform gyrus and parahippocampal gyrus when compared with those in healthy controls. Additionally, the WMV values of the bilateral putamen, left rectal gyrus, and thalamus were all increased in the patients with left-sided acoustic neuroma vs. those of healthy controls, yet no significant changes were found in the patients with right-sided acoustic neuroma. Furthermore, the GMV values of the left fusiform gyrus and parahippocampal gyrus and WMV values of the left rectal gyrus and precentral gyrus were higher in patients with left-sided acoustic neuroma than the patients with right-sided acoustic neuroma. No reductions were observed in the GMV and WMV values of the brain regions among all acoustic neuroma patients (Figures 4–7).

### Correlations of ALFF and ReHo values with cognitive function

As shown in Tables 3, 4, increased mALFF values could be observed in the right caudate nucleus of the patients with left-sided acoustic neuroma and the right rectal gyrus of the patients with right-sided acoustic neuroma, which were negatively correlated with cognitive function. On the contrary, the mReHo values of bilateral superior frontal gyrus and middle frontal gyrus were decreased, sharing positive correlations with cognitive function.

**Table 3:** Correlation analysis between mALFF and cognitive scale in patients with acoustic neuroma.

	**mALFF of the right caudate nucleus in LAN patients (*n =* 107)**	**mALFF of the right rectus gyrus in RAN patients (*n =* 91)**
MoCA scores	−0.373 (*p* < 0.001)^***^	−0.316 (0.002)^**^
Visuospatial executive	−0.338 (*p* < 0.001)^***^	−0.275 (0.008)^**^
Naming	−0.169 (0.081)	−0.215 (0.040)^*^
Attention	−0.127 (0.194)	−0.167 (0.113)
Language	−0.212 (0.028)^*^	−0.173 (0.100)
Language: sentence repetition	−0.192 (0.047)^*^	−0.157 (0.138)
Language: fluency task	−0.137 (0.159)	−0.080 (0.451)
Abstract thinking	−0.207 (0.032)^*^	−0.171 (0.105)
Delayed recall	−0.240 (0.013)^*^	−0.224 (0.032)^*^
Orientation	−0.088 (0.368)	−0.178 (0.090)
RAVLT immediate recall	−0.281 (0.003)^**^	−0.353 (0.001)^***^
RAVLT delay recall	−0.152 (0.119)	−0.244 (0.020)^*^
Stroop A (s)	0.342 (*p* < 0.001)^***^	0.295 (0.006)^**^
Stroop B (s)	0.179 (0.065)	0.273 (0.009)^**^
Stroop C (s)	0.217 (0.025)^*^	0.237 (0.024)^*^
SDMT	−0.291 (0.002)^**^	−0.234 (0.025)^*^
TMT A (s)	0.323 (0.001)^***^	0.236 (0.024)^*^
TMT B (s)	0.263 (0.006)^**^	0.249 (0.017)^*^

Data were expressed as spearman correlation coefficient (p-value).

* p < 0.05,

** p ≤ 0.01,

*** p ≤ 0.001; ALFF, Amplitude of low-frequency fluctuations; LAN, left acoustic neuroma; RAN, right acoustic neuroma; MoCA, Montreal cognitive assessment.

**Table 4:** Correlation analysis between mReHo and cognitive scale in patients with left acoustic neuroma.

	**mReHo value of left superior frontal gyrus (*n =* 107)**	**mReHo value of left middle frontal gyrus (*n =* 107)**	**mReHo value of right superior frontal gyrus (*n =* 107)**	**mReHo value of right middle frontal gyrus (*n =* 107)**
MoCA scores	0.133 (0.172)	0.201 (0.038)^*^	0.151 (0.120)	0.256 (0.008)^**^
Visuospatial executive	0.094 (0.336)	0.179 (0.065)	0.144 (0.138)	0.205 (0.034)^*^
Naming	0.084 (0.389)	0.024 (0.802)	0.044 (0.653)	0.112 (0.253)
Attention	0.210 (0.030)^*^	0.247 (0.010)^**^	0.211 (0.029)^*^	0.355 (*p* < 0.001)^***^
Language	0.202 (0.037)^*^	0.245 (0.011)^*^	0.269 (0.005)^**^	0.170 (0.081)
Language: sentence repetition	0.196 (0.043)^*^	0.268 (0.005)^**^	0.277 (0.004)^**^	0.132 (0.176)
Language: fluency task	0.090 (0.354)	0.060 (0.537)	0.137 (0.159)	0.177 (0.069)
Abstract thinking	0.081 (0.410)	0.159 (0.101)	0.155 (0.110)	0.106 (0.278)
Delayed recall	−0.036 (0.712)	0.020 (0.839)	−0.008 (0.932)	0.077 (0.433)
Orientation	0.169 (0.082)	0.131 (0.178)	0.063 (0.516)	0.320 (0.001)^***^
RAVLT immediate recall	0.152 (0.118)	0.186 (0.055)	0.140 (0.149)	0.185 (0.056)
RAVLT delay recall	0.123 (0.207)	0.127 (0.194)	0.108 (0.270)	0.174 (0.073)
Stroop A (s)	−0.125 (0.206)	−0.144 (0.146)	−0.032 (0.744)	−0.177 (0.072)
Stroop B (s)	−0.132 (0.175)	−0.172 (0.076)	−0.188 (0.053)	−0.216 (0.026)^*^
Stroop C (s)	−0.135 (0.164)	−0.186 (0.055)	−0.163 (0.093)	−0.198 (0.041)^*^
SDMT	0.088 (0.372)	0.157 (0.108)	0.013 (0.898)	0.129 (0.189)
TMT A (s)	−0.160 (0.099)	−0.200 (0.039)^*^	−0.084 (0.387)	−0.234 (0.015)^*^
TMT B (s)	−0.063 (0.520)	−0.122 (0.209)	−0.106 (0.279)	−0.236 (0.014)^*^

Data were expressed as spearman correlation coefficient (p-value).

* p < 0.05,

** p ≤ 0.01,

*** p ≤ 0.001; ReHo, regional homogeneity.

### Correlations of GMV and WMV values with cognitive function

**Figure 8:**
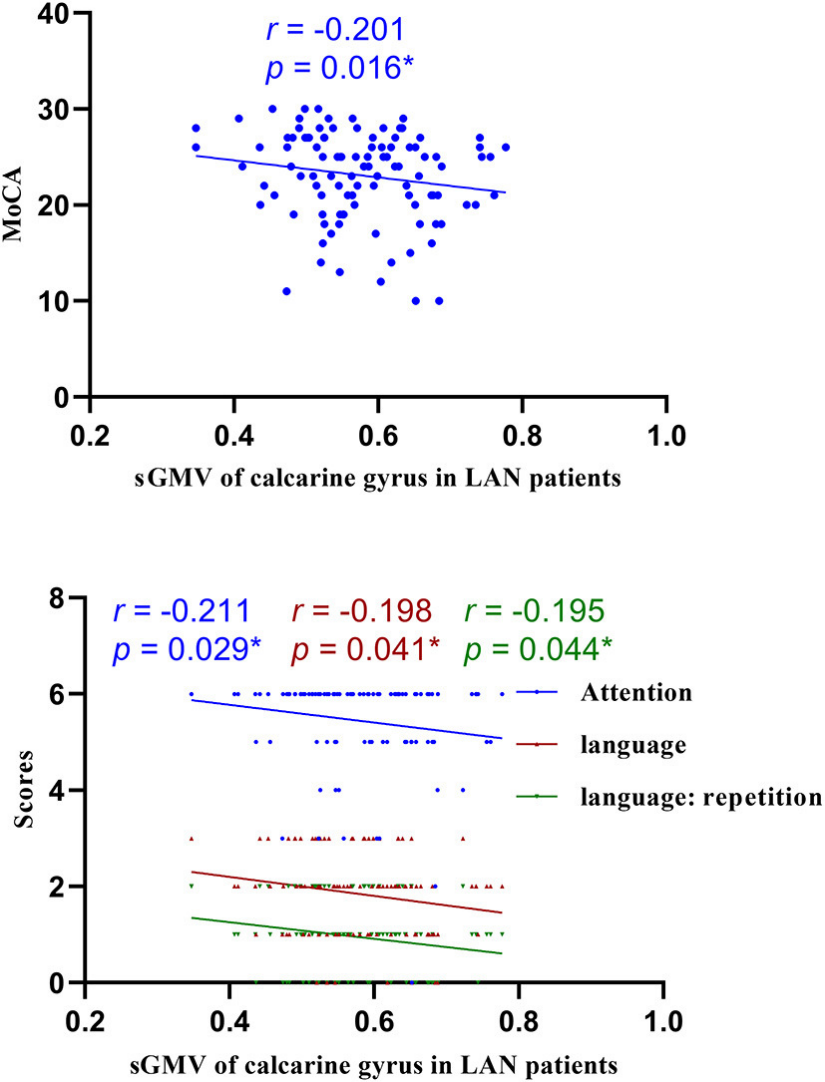
Correlations of sGMV of calcarine gyrus and MoCA in LAN patients. LAN, left acoustic neuroma; sGMV, smoothed gray matter volume. *p < 0.05.

**Figure 9:**
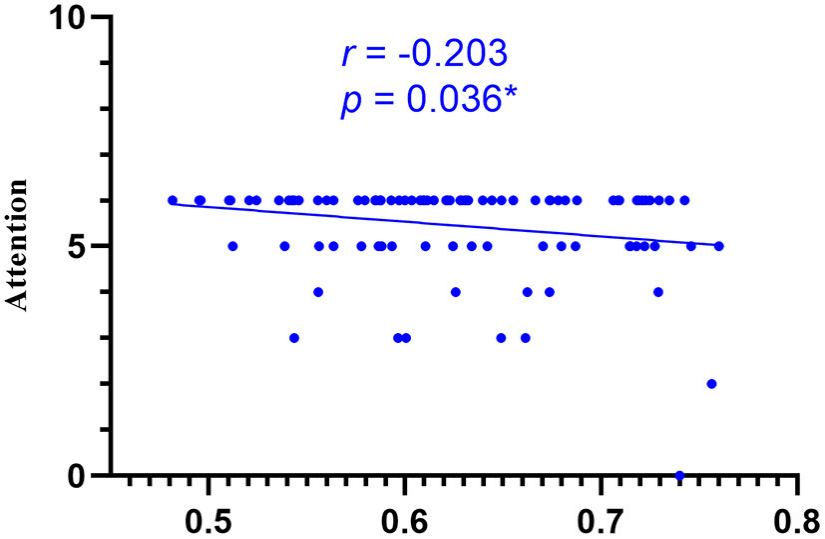
Correlations of sGMV of cuneus gyrus and attention of MoCA in LAN patients. LAN, left acoustic neuroma; sGMV, smoothed gray matter volume. *p < 0.05.

**Figure 10:**
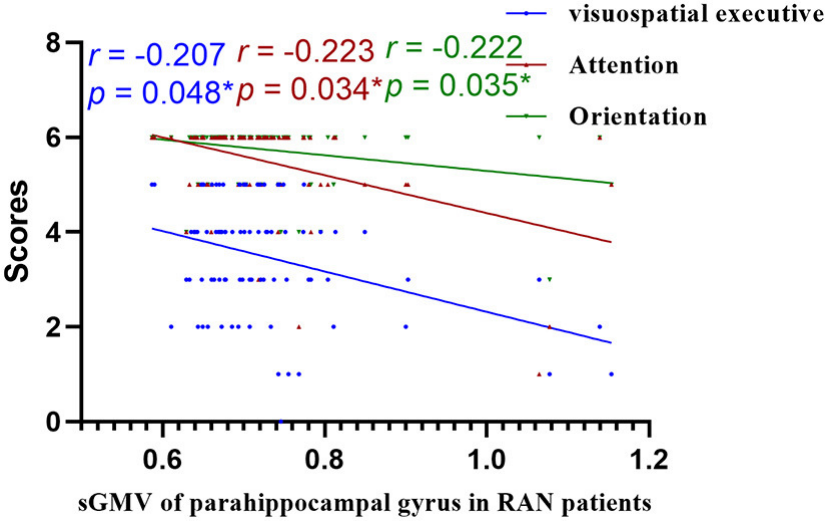
Correlations of sGMV of parahippocampal gyrus and MoCA in RAN patients. RAN, right acoustic neuroma; sGMV, smoothed gray matter volume. *p < 0.05.

We assessed the relevance of GMV and WMV values in the differential brain regions to the cognitive function scale and found that cognitive function was negatively correlated with the GMV and WMV values in the differential brain regions (Figures 8–10, Table 5).

**Table 5:** Results of the analysis of the correlation between differential white matter volume and cognitive and clinical information.

	**WMV of left putamen** **(*n =* 131)**	**WMV of right putamen** **(*n =* 131)**	**WMV of left rectus** **(*n =* 131)**	**WMV of left thalamus** **(*n =* 131)**
MoCA scores	−0.191 (0.029)^*^	−0.169 (0.054)	−0.256 (0.003)^**^	−0.126 (0.151)
visuospatial executive	−0.147 (0.095)	−0.157 (0.074)	−0.200 (0.022)^*^	−0.129 (0.140)
Naming	−0.128 (0.145)	−0.042 (0.638)	−0.057 (0.519)	0.020 (0.817)
Attention	−0.259 (0.003)^**^	−0.247 (0.004)^**^	−0.134 (0.127)	−0.194 (0.027)^*^
Language	−0.076 (0.389)	−0.117 (0.184)	−0.140 (0.111)	−0.025 (0.775)
Language: Sentence repetition	−0.054 (0.542)	−0.095 (0.282)	−0.123 (0.163)	−0.023 (0.795)
Language: fluency task	−0.068 (0.437)	−0.131 (0.137)	−0.162 (0.064)	0.070 (0.425)
Abstract thinking	−0.091 (0.300)	−0.080 (0.363)	−0.203 (0.020)^*^	−0.012 (0.888)
Delayed recall	−0.073 (0.407)	−0.044 (0.615)	−0.154 (0.079)	−0.094 (0.286)
Orientation	−0.183 (0.036)^*^	−0.173 (0.048)^*^	−0.193 (0.027)^*^	−0.118 (0.181)
RAVLT immediate recall	−0.262 (0.002) ^**^	−0.216 (0.013)^*^	−0.332 (*p* < 0.001)^***^	−0.176 (0.045)^*^
RAVLT delay recall	−0.261 (0.003)^**^	−0.25 (0.004)^**^	−0.265 (0.002)^**^	−0.156 (0.076)
Stroop A (s)	0.150 (0.095)	0.123 (0.171)	0.352 (*p* < 0.001)^***^	0.106 (0.240)
Stroop B (s)	0.113 (0.199)	0.155 (0.077)	0.252 (0.004)^**^	0.070 (0.425)
Stroop C (s)	0.148 (0.094)	0.137 (0.121)	0.323 (*p* < 0.001)^***^	0.041 (0.645)
SDMT	−0.083 (0.348)	−0.141 (0.110)	−0.331 (*p* < 0.001)^***^	0.017 (0.849)
TMT A (s)	0.132 (0.133)	0.234 (0.007)^**^	0.344 (*p* < 0.001)^***^	0.040 (0.647)
TMT B (s)	0.132 (0.132)	0.192 (0.028)^*^	0.323 (*p* < 0.001)^***^	0.069 (0.432)
Left PTA	0.204 (0.159)	−0.042 (0.777)	0.174 (0.232)	0.354 (0.013)^*^
Right PTA	0.077 (0.600)	0.284 (0.048)^*^	0.131 (0.370)	−0.112 (0.446)

Data were expressed as spearman correlation coefficient (p-value).

* p < 0.05,

** p ≤ 0.01,

*** p ≤ 0.001; PTA, pure tone average; WMV, white matter volume.

## Discussion

### Cognitive function of acoustic neuroma patients

In comparison with the healthy subjects, patients with left-sided acoustic neuroma and right-sided acoustic neuroma had unfavorable outcomes in the MoCA score, RAVLT memory, Stroop, SDMT, and TMT, showing a statistically significant difference. It is, therefore, illustrated that the cognitive function is declined in the patients with either left-sided or right-sided acoustic neuroma, and their memory, attention, executive function, motor speed, and information processing speed are greatly affected.

Previous studies have shown that children with UHL have worse language developmental outcomes and cognitive function than healthy children, corresponding to manifestations such as delayed language development and inattention (31), and the proportion of children with behavioral problems (25%) in which is higher than that of healthy children (32). Fan et al. (33) compared the cognitive function of patients with the right (*n* = 25) and left (*n* = 15) acoustic neuroma with healthy subjects, and their results revealed normality in the general cognitive function but declines in attention, information processing efficiency, executive function, and memory of the acoustic neuroma patients. Our study, not fully consistent with Fan's study, uncovered that in addition to the general cognitive function decline, significant impairments were induced in the attention, executive function, memory, visuospatial and visual perception abilities, motor speed, and information processing speed of patients with acoustic neuroma than healthy individuals. The acoustic neuroma patients included in this study showed worse neuropsychological test performance in more tasks, which is possibly attributed to our larger sample size and relatively high statistical power. Goebel et al. conducted their investigation on 27 patients with acoustic neuroma and 18 patients with meningioma, with the results demonstrating cognitive impairment in the majority of patients (69%), mainly manifested as decreased attention (such as alertness) and slowed visual motor speed (34), which is in agreement with our findings.

### Acoustic neuroma patients with tinnitus and cognition

Patients with acoustic neuroma are often associated with tinnitus. In our research, those patients accounted for 43.5% (30/69). To determine whether tinnitus interfered with the experiment, we tested the THI in patients with tinnitus symptoms and analyzed the correlation between neuropsychological tests and THI scores. The results showed that tinnitus did not affect cognitive function, anxiety, and depression, however, Chen et al. found that tinnitus affects cognitive function (35). We analyze the possible reasons as follows: first, the severity of tinnitus patients in the two experiments are different. Most of the tinnitus patients in their study are moderate and severe tinnitus of grade 3–4 (25/35, 71.43%), while most of our patients are mild tinnitus of grade 1–2 (29/30, 96.67%), and only 3.33% of moderate and severe tinnitus patients (1/30). Therefore, it may cause patient selection bias. The tinnitus symptoms of patients in our study are generally mild, which may be because patients are often not only complicated with symptoms such as tinnitus and hearing loss, but also headache, dizziness, and unstable walking, so it is more likely to attract the attention of patients and doctors, then examination found the existence of acoustic neuroma. Second, the heterogeneity of patients, our cases were acoustic neuroma with tinnitus symptoms, and their cases were patients with pure right tinnitus, so it may cause differences in experimental results. Third, the statistical power was limited due to the small number of tinnitus patients in the group, which also may have an impact on the experimental results. Meanwhile, Chen's study also found no correlation between tinnitus and anxiety or depression scale, which is consistent with our findings.

### Correlations of ALFF and ReHo parameters with the cognitive function of acoustic neuroma patients

Excavating brain activity is important for understanding the brain and gaining insights into its function (36). ALFF and ReHo are two robust indicators with defined physiological significance and good reproducibility in rs-fMRI studies. ALFF was first proposed by Zang et al. (27) that could represent the intensity of local brain activity. The BOLD signal contains time domain and frequency domain information, and ALFF reflects the strength of voxel spontaneous activity in the resting state by focusing on the frequency domain of the brain signal. ReHo was first proposed by Zang et al. (28) and applied to fMRI based on the assumption that the adjacent voxels in the same functional region have similar time-varying BOLD signals. ReHo describes the synchronization of adjacent voxel time series. A higher ReHo value indicates better synchronicity between adjacent voxels but does not describes stronger local neuron activity.

This study adopted rs-fMRI data to assess the changes in brain function parameters in patients with acoustic neuroma and analyze their relevance to cognitive function. The results indicated alterations in the mALFF and mReHo values of patients with acoustic neuroma. As compared to the healthy individuals, increases were found in the mALFF values of the right caudate nucleus and the right thalamus of the patients with left-sided acoustic neuroma as well as in the right rectal gyrus of the patients with right-sided acoustic neuroma, suggesting the possible existence of functional compensation in the brains of patients with acoustic neuroma and laterality biased to the right; meanwhile, the mReHo values of bilateral superior frontal gyrus and middle frontal gyrus were decreased in patients with left-sided acoustic neuroma, but no obvious changes were found in the right-sided acoustic neuroma group. Since ReHo reflects the synchronization of adjacent voxel time series, it was elucidated that the synchronicity of BOLD signals between the bilateral superior frontal gyrus and the middle frontal gyrus was decreased and the connectivity was declined, affecting the functional differentiation and integration of the brain, particularly the integration. This consequence may be linked to cognitive decline. The study of Wang et al. in the patients with left (*n* = 17) and right (*n* = 17) acoustic neuroma and healthy subjects (*n* = 22) displayed no difference in neurocognitive MMSE scores, but an increased ReHo value was presented in the left parahippocampal gyrus (37). They regarded it as compensation for cognitive decline. The conclusion of our experiments was different from those of Wang et al., and this difference may be associated with several factors. For instance, Wang's study enrolled younger subjects than our experiment, wherein the patients with left-sided acoustic neuroma had a mean age of 45.7 years, and those with right-sided acoustic neuroma had a mean age of 43 years; whereas, the actual age of patients in this experiment had a mean age of about 50 years. In Wang's study, the enrolled patients had a higher level of education and more severe hearing loss. Additionally, the two studies possibly involved the different stages of the body. Our study focused on the functional decline while Wang et al. mainly focused on the functional compensation, thus resulting in different results. Besides, the sensitivity of the MMSE adopted by Wang et al. was inferior to that of the MOCA we used here, which may also elicit no detectable difference.

In the present study, cognitive function was regarded to be reversely correlated with the mALFF value. We analyzed whether the body requires more resources to compensate for the decline in cognitive function owing to the cognitive function impairment of patients. The caudate nucleus, which belongs to basal nuclei, and the rectal gyrus are the two brain regions presenting increased mALFF. Previous studies have linked the basal nuclei to motor regulation, but multiple studies have currently reported the close relationship between the basal nuclei and cognitive function, including memory disorders and visuospatial disorders (38). Also, the rectal gyrus shares an association with memory (39). Therefore, decreased memory and executive control ability in patients can trigger the strengthened activity of some brain regions to compensate for the decline in cognitive function.

Decreased ReHo values of the bilateral superior frontal gyrus and middle frontal gyrus in patients with left-sided acoustic neuroma affected the general cognitive function (MoCA score), especially attention and language (including language repetition) in MoCA scores; meanwhile, the ReHo value of bilateral middle frontal gyrus was inversely related to the TMT, Stroop B, and C. The Stroop can be applied to evaluate the executive function and attention of subjects, and the TMT is employed to evaluate attention, executive control ability, and visuospatial ability. The prefrontal cortex is highly relevant to executive control and attention (40), which reflects a neuropathological basis supporting the cognitive function decline in patients with left-sided acoustic neuroma. However, there was no significant relation between the ReHo value on the right and the cognitive scale, firstly suggesting different impacts of left-sided and right-sided acoustic neuromas on the brain, which was consistent with the findings obtained in the previous sections. Secondly, the UHL caused by acoustic neuroma leads to lateralization trends in brain reorganization. Most of the results revealed significant differences between the acoustic neuroma and healthy control groups, but no significant difference existed between the left-sided and right-sided acoustic neuroma groups. Although no statistically significant difference was found in the current study, the reorganization phenomenon was more pronounced in patients with left-sided acoustic neuroma than in those with right-sided acoustic neuroma, possibly attributable to the stronger resistance of the right ear to hearing damage and its more stable behaviors (37, 41).

### Correlations of GMV and WMV parameters with the cognitive function of acoustic neuroma patients

Through VBM analysis of changes in the structures of gray and white matter in patients with acoustic neuroma, it was suggested that compared with healthy controls, patients with either left-sided or right-sided acoustic neuroma had increased GMV in several brain regions. Additionally, the WMV values in several brain regions were raised in the patients with left-sided acoustic neuroma, but none of the patients with right-sided acoustic neuroma showed brain regions with increased WMV. Also, none of the acoustic neuroma patients had reductions in the GMV and WMV values of the brain regions. Correlation analysis further revealed that the increases of GMV and WMV in acoustic neuroma patients were related to their attention, memory, executive function, etc.

Brain regions presenting an increased GMV contained the parahippocampal gyrus, fusiform gyrus, cuneus, and calcarine cortex, which are associated with functions such as computational ability, visuospatial ability, logical thinking, and memory (42). The parahippocampal gyrus is involved in memory formation (42). The fusiform gyrus, together with the middle and inferior temporal gyrus and the angular gyrus, participate in the formation of the temporoparietal language areas other than Wernicke's area, and together with the thalamus, represents the brain areas that are jointly activated by a sub-network (alertness) of the attention network and executive control network (40), which is involved in auditory semantic tasks, as well as meaning judgment of Chinese words, pictures, etc. (43). The cuneus and calcarine gyrus are related to functions such as visual function, calculation, and logical thinking (44). The increases in the volumes of the above-mentioned brain regions in patients with acoustic neuroma on the same side may be attributed to the reorganization of the brain structures to compensate for the decline in cognitive function. Different from our findings, Wang et al. (26) revealed atrophied cortical regions containing the bilateral anterior cingulate gyrus, the dorsolateral prefrontal cortex, the right superior frontal gyrus, and the bilateral middle frontal gyrus through comparisons among the patients with left-sided (*n* = 24) and right-sided (*n* = 24) acoustic neuroma and 24 normal subjects. They speculated that the aforementioned cognitive processing-related key structures may be related to cognitive dysfunction, but the MMSE score showed no difference. There are several differences between our study and the study of Wang et al., such as the age of the included patients, educational level, and neuropsychological test scales. The patients in this study had an average age of about 50 years, and those in Wang's study showed an average age of about 45 years. The patients with left-sided and right-sided acoustic neuroma of enrolled in Wang's study received about 12.3 and 10.7 years of education, respectively, and patients in this experiment underwent about 9.0 years of education. Additionally, we used a variety of neuropsychological tests with higher sensitivity, including MoCA, RAVLT, Stroop, SDMT, and TMT. The neuropsychological test results illustrated different degrees of cognitive impairment in the enrolled patients. However, Wang et al. only used MMSE for evaluation and did not find any difference between acoustic neuroma patients and healthy individuals. Whether the subjects included in the two experiments are in two different stages of the disease is still unclear, thus large-scale longitudinal studies for further clarification are demanded. Given the aforesaid differences, the conclusions driven by the two sets of experiments may not be the same. Based on our experimental data, we surmised that the long-term cognitive decline in patients with acoustic neuroma might lead to cortical reorganization to compensate for the cognitive decline.

It is not yet reported that WMV is altered following UHL, our study the first time pointed out that the areas such as bilateral putamen, left thalamus, and left rectal gyrus showed an increased WMV in acoustic neuroma patients. White matter constitutes an essential structure in the whole brain, and O'Sullivan et al. have substantiated that the integrity of white matter affects overall cognitive function and executive function (45). The putamen, belonging to the basal nuclei, principally regulates movement. A great number of studies have confirmed that the basal nucleus is highly relevant to cognitive impairment, such as memory impairment and visuospatial impairment (38). In addition to a close relation to consciousness and physical functions, the functions of the thalamus are often involved in the impairment of high-level cognitive functions such as memory, calculation, and language (46). Impairment of the gyrus rectus leads to impaired memory in patients (39). Our correlation analysis indicated that the attention, memory, and executive functions of patients with acoustic neuroma were inversely correlated with the WMV values of the putamen, gyrus rectus, and thalamus. We considered that body could compensate for the decreased cognitive function in the patients with cognitive impairment, leading to an increase in WMV in the aforementioned regions. In the meantime, we identified a positive correlation between left PTA and WMV in the left thalamus region and also a positive correlation between right PTA and the WMV in the right putamen. Higher severity of the PTA correlated with worse hearing. Whether the increased volume of white matter in the left thalamus region and right putamen are jointly induced by hearing loss and cognitive decline remains still unclear, and is necessary to be validated through additional animal studies.

The changes in GMV and WMV in left-sided and right-sided acoustic neuromas were not completely consistent. Relatively smaller reorganization regions of brain structure can be induced by the right-sided acoustic neuroma, which is likely linked to the different effect mechanisms of the left-sided and right-sided acoustic neuromas on the body. This conclusion has been repeatedly substantiated by the experimental results in the previous sections. Some scholars believe that the right ear has enhanced resistance to hearing damage with higher stability (37). The left ear shows a stronger contralateral effect in the process of monaural reception of sound stimulation, while the right ear transmits the information evenly to both hemispheres. Once the acoustic neuroma interferes with normal patterns, the dysfunction of the left ear will induce a more pronounced disturbance to and affect the brain. GMV and WMV in patients with acoustic neuroma exhibited lateral changes, which were ipsilateral to the lesion. Thus, the compensatory mechanism of the body after the cognitive decline in patients often occurs on the same side of the lesion. Acoustic neuroma exhibits plasticity in the cognitive representation system, presenting anatomically distinguishable structural reorganizations to compensate for cognitive impairment resulting from impaired auditory input.

## Conclusion

Cognitive dysfunction in patients with acoustic neuroma encompasses general cognitive function, executive function, attention, visuospatial executive ability, memory, visual perception ability, motor speed, information processing speed, etc. Changes can be found in the markers ALFF and ReHo in acoustic neuroma patients. Cognitive decline in the patients with acoustic neuroma activates functional activity in some brain regions, thereby compensating for the decline in cognitive function. In the meantime, the reductions in the ReHo values and connectivity of the bilateral superior frontal gyrus and middle frontal gyrus of the patients with lateral acoustic neuroma may affect the functional differentiation and integration of the brain, which is likely related to the cognitive function decline. Brain reorganization induced by UHL in patients with acoustic neuroma exhibits lateralization trends. Left-sided acoustic neuroma induces a more significant influence on the brain, and right-sided acoustic neuroma shows a more stable performance of the cerebral cortex. The cognitive function of patients with acoustic neuroma declines, the body undergoes structural reorganization, and the GMV and WMV values are increased compensatively in the cognitive-related brain regions to compensate for cognitive impairment.

## Data availability statement

The raw data supporting the conclusions of this article will be made available by the authors, without undue reservation.

## Ethics statement

The studies involving human participants were reviewed and approved by Ethics Committee of Nanchong Central Hospital. The patients/participants provided their written informed consent to participate in this study. Written informed consent was obtained from the individual(s) for the publication of any potentially identifiable images or data included in this article.

## Author contributions

XD, LizL, and ZZ designed the study and collected the data. XD, QC, and LihL analyzed the data. XD wrote the paper. ZZ, LihL, and XH drafted the paper. All authors contributed to the article and approved the submitted version.

## Funding

This work was supported by Bureau of Science and Technology Nanchong City (Grant Nos. 19SXHZ0273 and 20YFZJ0115), Nanchong Social Science Federation (Grant No. NC21B188), Sichuan Province Medical Youth Innovative Research Project Program (Grant No. Q21029), Primary Health Development Research Center of Sichuan Province (Grant No. SWFZ20-C-069), and Special Funding for Postdoctoral Research Projects of Chongqing (Grant No. 2021XM3012).

## Conflict of interest

The authors declare that the research was conducted in the absence of any commercial or financial relationships that could be construed as a potential conflict of interest.

## Publisher's note

All claims expressed in this article are solely those of the authors and do not necessarily represent those of their affiliated organizations, or those of the publisher, the editors and the reviewers. Any product that may be evaluated in this article, or claim that may be made by its manufacturer, is not guaranteed or endorsed by the publisher.

## Abbreviations

AN: acoustic neuroma
HC: healthy control
LAN: left acoustic neuroma
MoCA: Montreal cognitive assessment
PTA: pure tone average
RAN: right acoustic neuroma
RAVLT: Rey Auditory Verbal Learning Test
SDMT: symbol-digital modalities test
TMT: trail-making test
UHL: unilateral hearing loss
ALFF: amplitude of low-frequency fluctuations
ReHo: regional homogeneity
VBM: voxel-based morphometry
GMV: gray matter volume
WMV: white matter volume.
