# Supplementary material for: Cognitive decline in acoustic neuroma patients: An investigation based on resting-state functional magnetic resonance imaging and voxel-based morphometry
Source: Front Psychiatry. 2022 Aug 1;13:968859. doi: 10.3389/fpsyt.2022.968859 (PMC9376325; doi:10.3389/fpsyt.2022.968859)
Supplement: Supplementary file 1 [file Data_Sheet_1.docx]

**Supplementary Table 1 Demographic and clinical characteristics of AN and HC**

|  | AN（*n=*64） | HC（*n=*67） | Z/T values | | *P* value |
| --- | --- | --- | --- | --- | --- |
| gender(male) | 21（32.8%） | 21（31.3%） | 0.032 | 0.857^a^ | |
| age(yrs) | 49.56±13.51 | 45.75±10.77 | 1.792 | 0.075^b^ | |
| years of education(yrs) | 9.00（6.8） | 9.00（7.00） | -0.763 | 0.446^c^ | |
| Course of disease(yrs) | 2.00（5.46） | N/A | N/A | N/A | |
| Left PTA (dB HL) | 37.50（38.75） | N/A | N/A | N/A | |
| Right PTA (dB HL) | 21.25（35.00） | N/A | N/A | N/A | |
| THI | 11.00（6.00） | N/A | N/A | N/A | |
| MoCA | 21.00（8.00） | 26.00（5.00） | -5.956 | <0.001^c*^ | |
| RAVLT immediate recall | 33.00（16.00） | 47.00（19.00） | -5.644 | <0.001^c*^ | |
| RAVLT delay recall | 6.00(5.00) | 9.00(5.00) | -4.964 | <0.001^c*^ | |
| Stroop A(s) | 33.00（22.00） | 27.00（12.50） | -3.084 | 0.002^c*^ | |
| Stroop B(s) | 49.50（30.00） | 37.00（19.00） | -3.989 | <0.001^c*^ | |
| Stroop C(s) | 130.00（78.00） | 86.00（52.00） | -4.689 | <0.001^c*^ | |
| SDMT | 33.84±16.19 | 45.16±15.84 | -4.030 | <0.001^b*^ | |
| TMT A(s) | 58.50（69.00） | 38.00（31.00） | -4.292 | <0.001^c*^ | |
| TMT B(s) | 192.00（190.00） | 104.00（111.00） | -4.002 | <0.001^c*^ | |
| HAMD | 9.00（7.00） | 2.00（3.00） | -8.053 | <0.001^c*^ | |
| HAMA | 6.50（6.00） | 2.00（2.00） | -7.804 | <0.001^c*^ | |

^a^*P* and ^b^*P* were obtained by chi-square test and t-test (two-tailed), respectively. ^c^*P* obtained by Mann-Whitney U nonparametric test. Z values and T values were obtained by nonparametric test and t-test, respectively. All data are presented as mean ± SD, median (interquartile range), or number (percentage). The significance level was set at *P* < 0.05. **P* <0.05. AN: acoustic neuroma. HC: healthy controls. PTA: pure tone average; N/A: not available.

**Supplementary Table 2 Analysis of the effects of AN and HC on MoCA**

|  | AN（*n=*64） | HC（*n=*67） | Z values | *P* value |
| --- | --- | --- | --- | --- |
| visuospatial executive | 2.50（1.00） | 4.00（2.00） | -6.585 | <0.001^*^ |
| naming | 3.00（2.00） | 3.00（1.00） | -1.891 | 0.059 |
| attention | 5.00（1.00） | 6.00（0.00） | -5.228 | <0.001^*^ |
| language | 1.00（1.00） | 2.00（2.00） | -4.210 | <0.001^*^ |
| language: Sentence repetition | 0.00（1.00） | 1.00（1.00） | -3.671 | <0.001^*^ |
| language: fluency task | 1.00（0.00） | 1.00（0.00） | -3.820 | <0.001^*^ |
| abstract thinking | 1.00（2.00） | 2.00（1.00） | -3.016 | 0.003^*^ |
| delayed recall | 2.00（2.00） | 3.00（3.00） | -3.664 | <0.001^*^ |
| orientation | 6.00（1.00） | 6.00（0.00） | -3.408 | 0.001^*^ |
| MoCA scores | 21.00（8.00） | 25.50（4.00） | -5.956 | <0.001^*^ |

Z and *P* values were obtained by Mann-Whitney U nonparametric test. Data are presented as median (interquartile range). * *P* <0.05。AN: acoustic neuroma. HC: healthy controls.


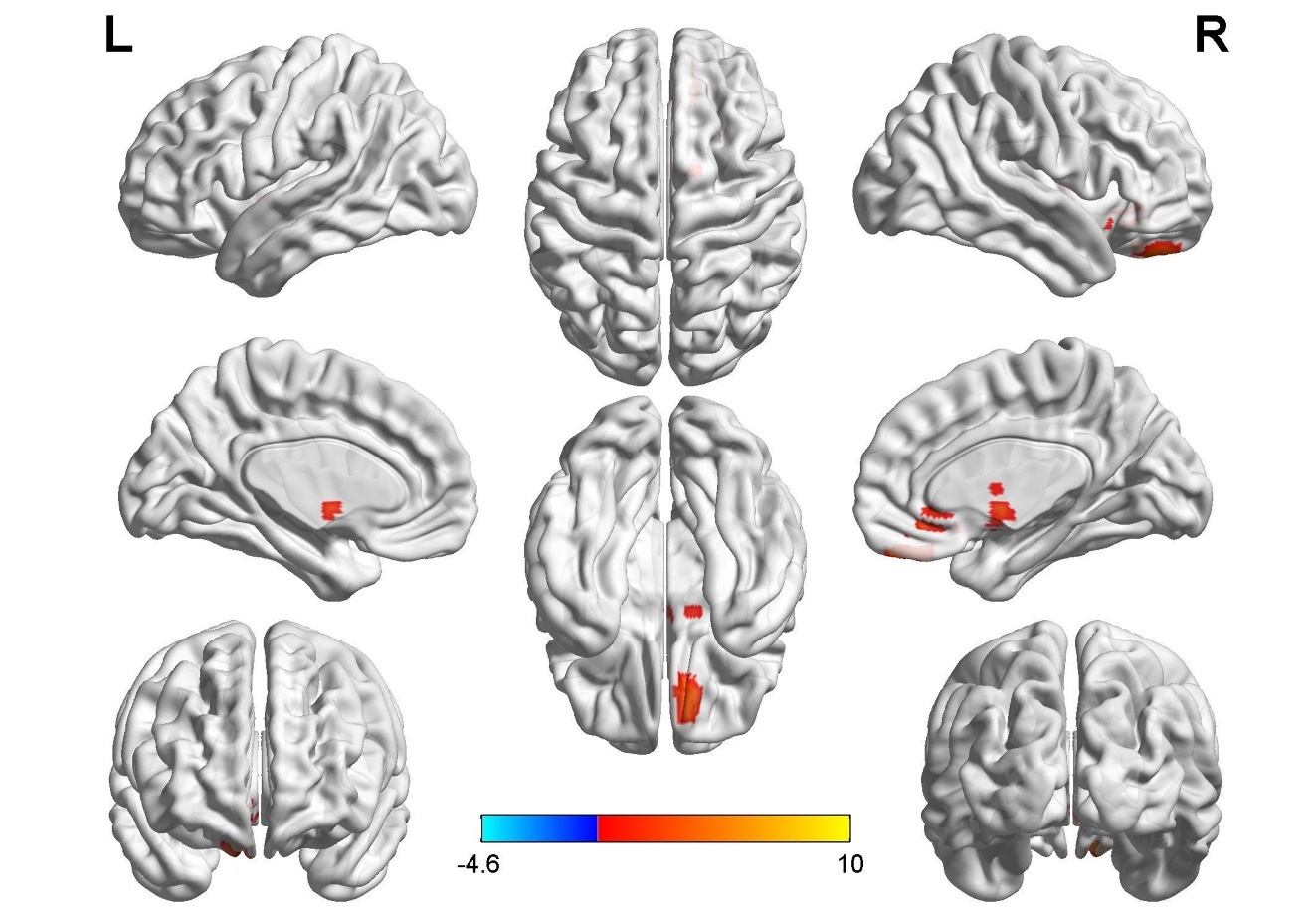
 **Supplementary Figure 1 T-value map of the differential mALFF results of ANOCOVA among left and right auditory neuroma and healthy controls.**

Subjects in the three groups showed increased mALFF values in the right caudate nucleus and right rectus gyrus, with no significant decrease in the brain regions. The statistical threshold was voxel-wise *P* < 0.001 with cluster-wise FWE corrected *P* < 0.05 (124 voxels).
